# Perioperative Management of Congenital Diaphragmatic Hernia Repair in a Patient With Univentricular Circulation: A Case Report

**DOI:** 10.7759/cureus.33338

**Published:** 2023-01-04

**Authors:** Natsuki Suzuki, Hisakatsu Ito, Shota Sakai, Katsuhisa Hirano, Kentaro Tamura

**Affiliations:** 1 Department of Anesthesiology, Faculty of Medicine, Academic Assembly, University of Toyama, Toyama, JPN; 2 Department of Anesthesiology, Toyama University Hospital, Toyama, JPN; 3 Department of Surgery and Science, Faculty of Medicine, Academic Assembly, University of Toyama, Toyama, JPN; 4 Division of Neonatology, Maternal and Perinatal Center, Toyama University Hospital, Toyama, JPN

**Keywords:** perioperative management, pulmonary hypoplasia, univentricular heart, congenital heart disease, congenital diaphragmatic hernia

## Abstract

The survival rate in congenital diaphragmatic hernia (CDH) with complex heart defects is low. Although the current consensus on the indications for surgical repair of CDH without heart defects has improved surgical outcomes, the surgical indication for CDH with complex heart defects remains unclear. Herein, we report the perioperative management of a patient with univentricular circulation who underwent CDH repair. Thus, patients with CDH complicated by univentricular anatomy may tolerate surgery depending on preserved respiratory function.

## Introduction

Congenital diaphragmatic hernia (CDH) is a fatal disease characterized by defective diaphragm formation, pulmonary hypoplasia, and pulmonary hypertension that requires surgical repair. Single ventricle (SV) is a cyanotic congenital heart disease that needs a parallel supply of pulmonary and systemic circulations. The surgical prognosis of CDH has improved since the introduction of the consensus which clarifies the indication for surgery in CDH EURO Consortium [1]. However, the survival rate in CDH patients with complex heart defects is low, at approximately 36%, and with SV is particularly low at approximately 16% [2], and the indications for surgery remain unclear. One reason for this is the complexity of the pathophysiology, in which pulmonary hypoplasia and hypertension in CDH make it difficult to maintain a balance between pulmonary and systemic circulation. Herein, we present the perioperative management of a two-day-old infant with univentricular circulation for bilateral CDH repair. This article was previously presented as an oral presentation at the 2022 Japan Society for Clinical Anesthesia on November 11, 2022.

## Case presentation

A two-day-old female born at 38 weeks of gestation weighing 2 kg, classified in American Society of Anesthesiologists (ASA) class Ⅳ, was prenatally diagnosed with trisomy 18, CDH, and congenital heart disease, including mitral atresia, double-outlet right ventricle, pulmonary stenosis, and severe tricuspid regurgitation. She was immediately intubated at birth due to generalized cyanosis and bradycardia.

**Figure 1:**
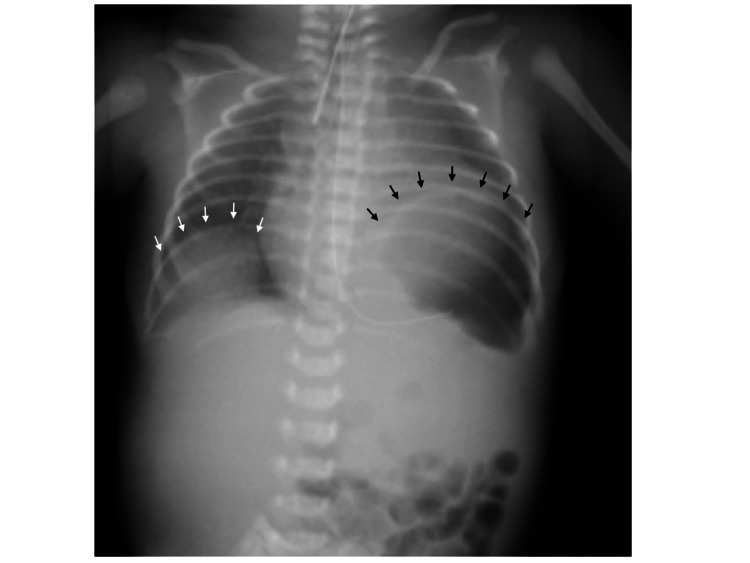
Preoperative chest X-ray Preoperative anteroposterior X-ray shows bowel gas in the left thorax (black arrows) and partial elevation of the diaphragm in the right thorax (white arrows).

Chest radiography revealed bowel gas in the left thorax and a partially elevated diaphragm in the right thorax (Figure 1). Echocardiography revealed that the pulmonary blood flow depended on the patent ductus arteriosus (PDA), and prostaglandin E1 was started at 3 ng/kg/min. The atrial septal defect was 7 mm and unrestrictive. Fentanyl was administered at 1 µg/kg/h in NICU with sedation. She was ventilated using pressure control ventilation (PCV) with a peak inspiratory pressure (PIP) of 20 cmH_2_O, resultant tidal volume (TV) of 8-12 mL, respiratory rate (RR) of 40 breaths/min, and fraction of inspired oxygen (FiO_2_) of 21-30%. Arterial blood gas (ABG) revealed pH 7.354, partial pressure of oxygen (PaO_2_) 40.2 mmHg, partial pressure of carbon dioxide (PaCO_2_) 43.0 mmHg, base excess (BE) -1.7 mmol/L, and lactate 1.5 mmol/L. Hemodynamics were stable without inotropic support, with a BP of 53/34 mmHg, HR of 157 beats/min, and oxygen saturation (SpO_2_) of approximately 90%. Surgical repair of the CDH was scheduled two days after birth.

An invasive arterial pressure catheter had been inserted in the NICU. Inhaled nitric oxide (iNO), high-frequency oscillatory ventilation (HFOV), and extracorporeal membrane oxygenation (ECMO) were on standby. After slow induction with sevoflurane via the existing endotracheal tube, rocuronium was administered. PCV was initiated with a PIP of 22 cmH_2_O, RR of 35 breaths/min, and FiO_2_ of 30%. A central venous catheter was placed in the right internal jugular vein under ultrasonographic guidance. Olprinone (0.1 µg/kg/min), along with dopamine and dobutamine (1 µg/kg/min each), was initiated. Anesthesia was maintained with a low concentration of sevoflurane (1.0-1.5%) and continuous infusion of fentanyl (10 µg/h) and midazolam (0.2 mg/h). Bilateral diaphragmatic hernia with sacs was confirmed after a transverse abdominal incision. The sacs were resected and directly sutured. Albumin and red blood cell transfusions were required because of hypotension caused by hemorrhage and insensible perspiration. CVP < 15 cmH2O was maintained to prevent infusion overload, and dopamine and dobutamine doses were increased to 5 µg/kg/min, aiming for at least preoperative vital signs. Surgical maneuvers sometimes inhibited ventilation; ABG revealed pH 7.117, PaO_2_ 66.9 mmHg, PaCO_2_ 69.0 mmHg, BE -8.0 mmol/L, and lactate 1.9 mmol/L after suturing the left diaphragm. Acidosis progressed after suturing the right diaphragm. The ventilator settings were adjusted with a PIP < 25 cmH2O and RR < 40 breaths/min. Furthermore, SpO_2_ was maintained at approximately 90% by adjusting FiO2 to 30-50% during the procedure. Transfusion was administered as a bolus with a target hematocrit of 35-45% to compensate for metabolic acidosis associated with hypovolemia. Adrenaline was administered at 0.02 µg/kg/min for persistent hypotension. Thoracic drains were placed bilaterally, and the surgery was completed without severe complications. Respiratory status showed no significant change, and hemodynamics gradually stabilized after wound closure.

She was transported to the NICU with stable respiratory and hemodynamic parameters and ventilated with FiO_2_ 30%. The patient underwent pericardial drainage for cardiac tamponade on postoperative day one. Furthermore, HFOV was initiated due to respiratory failure associated with pulmonary edema on postoperative days four to seven. Prostaglandin E1 was tapered off on postoperative day three because PDA had caused excessive pulmonary blood flow. As urine output increased, respiratory status stabilized, and inotropes were tapered off. 

## Discussion

Permissive hypercapnia and ‘gentle ventilation’ can increase the survival rate of neonates with CDH. In general, the CDH EURO Consortium recommends limiting PIP to < 25 cmH_^2^_O, positive end-expiratory pressure (PEEP) to 3-5 cmH_2_O, and adjusting ventilator rate to obtain PaCO_2_ between 50 and 70 mmHg [1]. In our case, the patient could maintain ‘gentle ventilation’ during surgery. Intraoperative fluid overload was avoided by CVP monitoring and inotrope use to prevent postoperative pulmonary edema. Consequently, the patient was able to overcome postoperative respiratory failure with temporary HFOV. Traditionally, inhalation anesthetics with mechanical ventilation, opioids, and muscle relaxants are used for CDH repair surgery. However, total intravenous anesthesia could have better potential because inhaled anesthetics are difficult to apply with HFOV and have cardiac suppression and airway irritation with high concentration [3].

The consortium recommends a surgical repair after physiological stabilization; normal mean arterial pressure (MAP) for gestation, preductal SpO_2_ 85-95% (FiO_2_ $\leq$ 50%), lactate $\leq$ 3 mmol/L, and urine output $\geq$ 1 ml/kg/h [1]. A facility in Ireland has established the following criteria for surgical indications; SpO_2_ $\geq$ 92% (FiO_2_ $\leq$ 50%), MAP $\geq$ 45 mmHg (noradrenaline and adrenaline < 0.05 µg/kg/min), pulmonary artery pressure < 2/3 systemic pressure, weaning iNO ($\leq$ 10 ppm), and hemoglobin $\geq$ 10 g/dL [4]. Since our case met most of the above criteria except for low BP, the surgical decision was reasonable. Whether 18 trisomy has a surgical indication for CDH is controversial. In our institution, CDH repair for patients with 18 trisomy is considered if the family desires surgery after discussing its advantages and disadvantages. In our case, CDH repair was effective regarding respiratory setting and enteral nutrition.

Congenital heart defects are detected in 10-35% of patients with CDH and have a poor prognosis. The prognosis could be due to the reluctance to undergo surgery owing to the increased complexity of patients due to multi-organ involvement [2]. Previous studies have reported a reduced survival rate of 36% in CDH patients with complex heart disease, particularly low SV (approximately 16% presenting SV) [2]. Generally, SV needs SpO_2_ 75-85% to balance the pulmonary and systemic circulation [5]. SV also requires maintaining a high hematocrit to preserve oxygen supply. In contrast, an excessively high hematocrit is not preferred because it may increase pulmonary vascular resistance in both CDH and SV. Pulmonary hypertension due to pulmonary hypoplasia and high pulmonary vascular resistance is common in CDH and unfavorable for SV because of the difficulty in maintaining parallel circulation. In our case, bilateral sac-type CDH limited the abdominal organ deviation. The preoperative respiratory status was stable without a high oxygen concentration or HFOV, even though pulmonary blood flow depended on PDA. This indicated that the pulmonary hypoplasia and hypertension were not severe, which could be one of the reasons that the patient tolerated the repair surgery.

The perioperative SpO_2_ of 90% could be high for SV. The preoperative BP was the lower limit of normal for neonates, suggesting that the patient was more likely to flow into the pulmonary circulation than into the systolic circulation. The large diameter of PDA and relatively low pulmonary vascular resistance could cause excessive pulmonary blood flow. The patient did not undergo cardiac catheterization; the detailed hemodynamics and Qp/Qs ratio remain unknown. In this case, a lower SpO_2_ target might have made it easier to maintain BP because of the increased volume of circulating systemic blood. iNO is usually the first-line therapy for treating pulmonary hypertension in infants with CDH [1]. In our case, iNO was not necessary throughout the perioperative period; rather, iNO could exacerbate high pulmonary flow.

## Conclusions

Patients with CDH with congenital heart defects have a poor prognosis. Uncertainty regarding the indications for surgery has been suggested as one of the reasons. This case report suggests the possibility of tolerance for surgery in patients with CDH presenting with univentricular circulation in cases of preserved respiratory function.

## References

[1] Snoek KG, Reiss IK, Greenough A (2016). Standardized postnatal management of infants with congenital diaphragmatic hernia in Europe: the CDH EURO Consortium Consensus - 2015 Update. Neonatology.

[2] Menon SC, Tani LY, Weng HY, Lally PA, Lally KP, Yoder BA (2013). Clinical characteristics and outcomes of patients with cardiac defects and congenital diaphragmatic hernia. J Pediatr.

[3] Hsu HT, Lin JY, Tseng HI (2004). Total intravenous anesthesia for repair of congenital diaphragmatic hernia: a case report. Kaohsiung J Med Sci.

[4] Fennessy P, Crowe S, Lenihan M, Healy M (2018). Anesthesia consensus on clinical parameters for the timing of surgical repair in congenital diaphragmatic hernia. Paediatr Anaesth.

[5] Rao PS (2021). Single ventricle-a comprehensive review. Children (Basel).

